# Unlocking the therapeutic potential of thymus-isolated regulatory T cells

**DOI:** 10.3389/fimmu.2025.1612360

**Published:** 2025-06-26

**Authors:** Yueyuan Hu, Hugo Cruz, Samikshya Santosh Nirmala, Anke Fuchs

**Affiliations:** ^1^ Center for Regenerative Therapies Dresden (CRTD), Center for Molecular and Cellular Bioengineering (CMCB), TUD Dresden University of Technology, Dresden, Germany; ^2^ DKMS Stem Cell Bank, Deutsche Knochenmarkspenderdatei (DKMS), Dresden, Germany

**Keywords:** regulatory T cells, thymus, thyTregs, Treg isolation, *in vitro* Treg expansion, Treg cell therapy, ex vivo Treg expansion

## Abstract

Regulatory T cells (Tregs) play a pivotal role in modulating excessive immune responses and maintaining immune homeostasis in humans. Notably, therapeutic strategies employing autologous and allogeneic Tregs have shown promising signs of efficacy in the treatment and prevention of graft versus-host disease (GvHD), transplant rejection and autoimmune diseases. Treg cells are typically obtained from peripheral blood or umbilical cord blood, but the largely antigen-experienced memory state of peripheral blood Tregs and the limited number of Tregs that can be isolated from cord blood remain obstacles. However, recent studies have identified the thymus as a novel and promising source of Tregs, overcoming the abovementioned limitations. Currently, human thymus-isolated regulatory T cells (thyTregs) are being investigated in phase 1/2 clinical trials to assess their safety and efficacy in both autologous and allogeneic settings. This review provides a comprehensive overview of the different manufacturing processes for isolation and expansion of thymus-derived regulatory T cells, their clinical relevance and current ongoing clinical trials investigating the therapeutic potential of this novel class of Tregs.

## 1 Introduction

### 1.1 Treg function

Regulatory T cells (Tregs) are essential regulators of immune tolerance to prevent autoimmunity. They are capable of suppressing exacerbated immune responses under proinflammatory conditions (1), as well as promoting tissue homeostasis and repair after inflammation (2–4). Due to their highly suppressive capacity, CD4^+^CD25^+^FOXP3^+^ Tregs play crucial roles in the control of autoimmunity and tolerance induction in transplantation (5–7). On the other hand, Treg deficiency or dysregulation can lead to several autoimmune diseases, including multiple sclerosis, type 1 diabetes (T1D), systemic lupus erythematosus, rheumatoid arthritis, and psoriasis (8–12).

Tregs can suppress the activity of several immune cells, such as CD4^+^ and CD8^+^ T effector cells (Teffs), B cells, dendritic cells (DC), macrophages, granulocytes, natural killer cells and osteoclasts (13), through several direct or indirect mechanisms (14–17). The main suppressive strategies include: interference with antigen presentation, production of immunosuppressive cytokines (including IL-10, IL-35 and TGF-β), consumption of cytokines and growth factors such as IL-2, essential for Teff activation, development and proliferation, and also metabolic disruption to decrease Teff cells activity (14, 18–20).

### 1.2 Treg development in the thymus

Although Treg can be induced in the periphery from naïve conventional T cells, Treg development primarily occurs in the thymus. It begins when bone marrow-derived thymic seeding progenitors (TSPs) enter the thymus and progress through several stages: double negative (CD4^-^CD8^-^, DN), double positive (CD4^+^CD8^+^, DP), and finally single positive (CD4^+^CD8^-^ or CD4^-^CD8^+^, SP) stages (21, 22). DN thymocytes are further classified into DN1 to DN4 based on CD44 and CD25 expression (23, 24). Notch signaling in early thymic progenitors (DN1) drives T cell lineage commitment, marked by the upregulation of CD25 and progression to the DN2a stage (25, 26). As thymocytes develop, they undergo T cell receptor (TCR) gene rearrangement followed by a rigorous selection process based on their ability to bind to the self-peptide/self-MHC (major histocompatibility complex) (27, 28). The cells with low affinity (~90-96% of thymocytes) and cells with very high affinity (~2-5% of thymocytes) undergo death by neglect or deletion through negative selection, whereas cells with intermediate affinity are further selected to become mature CD4 and CD8 SP cells (29). A very small portion (<1% of thymocytes) with intermediate to high affinity are selected to become Tregs (30, 31).

**Figure 1:**
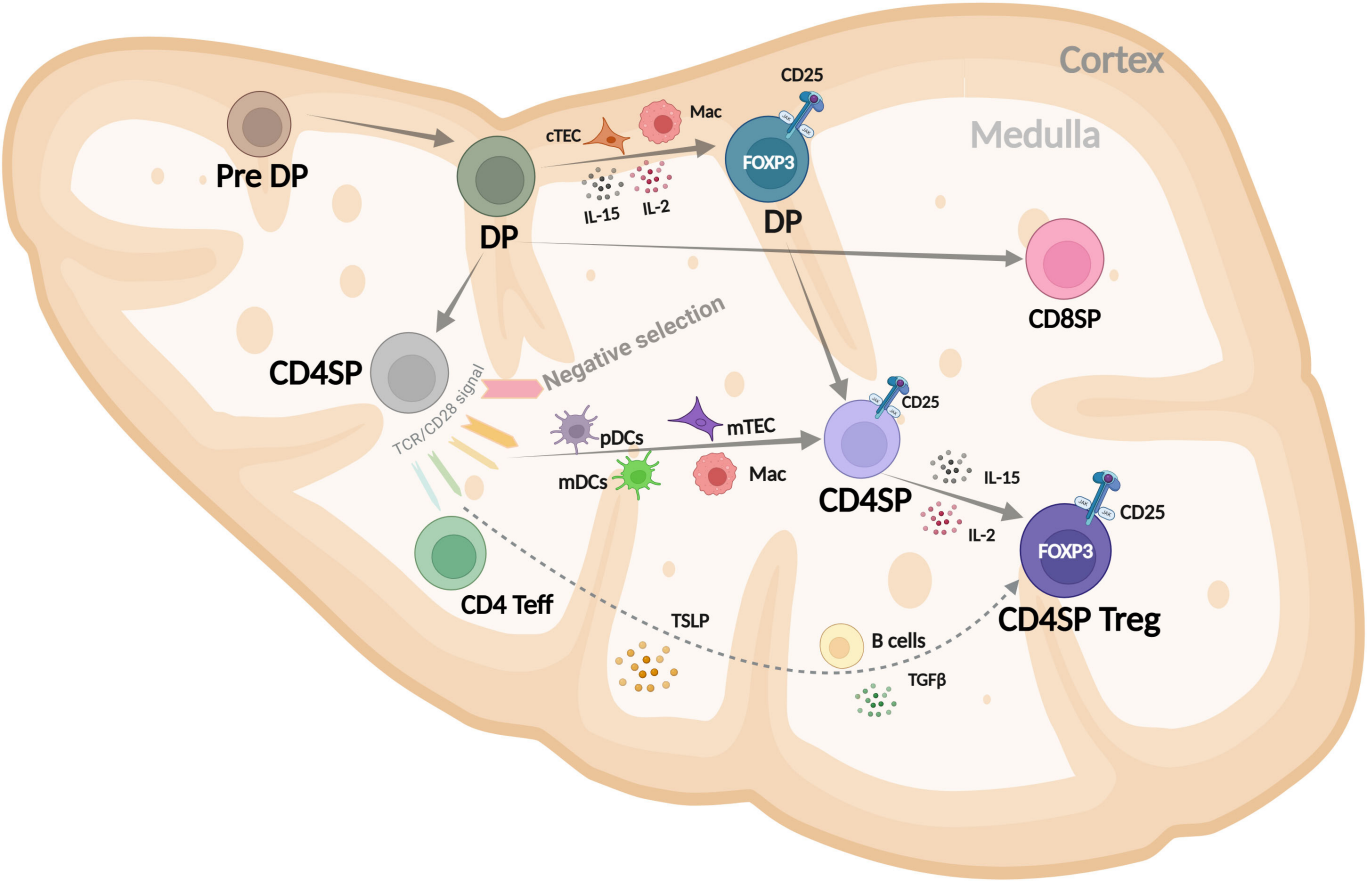
Schematic representation of human CD4^+^ Treg development in the thymus, highlighting key stages and signals in the cortex and medulla that drive FOXP3 expression and lineage commitment. DP, double positive (CD4^+^CD8^+^); SP, single positive (CD4^+^CD8^-^); cTEC, cortical thymic epithelial cells; Mac, macrophage; mDC, myeloid dendritic cell; pDC, plasmacytoid dendritic cell; TSLP, thymic stromal lymphopoietin. Modified from Caramalho I et al. (32).

Most of our understanding of Treg development comes from mouse models. In humans, however, technical limitations and the lack of specific tools have made it challenging to fully define how Tregs develop. Despite this, some insights into human thymic Treg (thyTreg) development have been reported (32, 33). Current evidence suggests that Treg lineage commitment in humans may occur at multiple stages of T cell development as illustrated in **Figure 1**.

The most immature thymocyte population in humans that clearly expresses FOXP3, along with key Treg markers like CD25, CTLA-4, and CD39, and displays regulatory function is the cortical CD4^+^CD8^+^ DP subset (34–36). Their positive selection and commitment to the Treg lineage are likely influenced by cortical thymic epithelial cells (cTECs), macrophages, and local cytokines such as IL-2 and IL-15, which are present in the thymic cortex (37). The FOXP3^+^ DP cells strongly correlate with the FOXP3^+^ CD4SP population, suggesting a precursor–product relationship (36). Medullary CD4SP FOXP3^-^ thymocytes can upregulate FOXP3 and become Tregs after interacting with activated plasmacytoid or myeloid dendritic cells (pDCs or mDCs) in a process that depends on IL-2. These FOXP3^-^ thymocytes can receive TCR and costimulatory signals that induce CD25 expression, giving rise to Treg precursors (CD4SP CD25^+^FOXP3^-^) (37–39). These precursors can then differentiate into thymic Tregs in response to IL-2 -mainly from proliferating CD4SP thymocytes- or IL-15 secreted by macrophages, B cells, or medullary thymic epithelial cells (mTECs). It is also possible that simultaneous TCR, costimulatory, and γ-chain cytokine signaling directly drives FOXP3 expression in CD4SP FOXP3^-^ cells, but this needs further investigation. Additionally, CD4SP CD25^-^FOXP3^-^ thymocytes can acquire FOXP3 and become Tregs when stimulated through the TCR in the presence of costimulation, TGF-β, and IL-2 or IL-15 (37, 40).

### 1.3 Treg cell therapy

The efficacy of Treg-based therapies has initially been demonstrated in several murine models over the last two decades (41–46). Later, clinical applications utilizing Tregs as adoptive cell therapy have demonstrated safety (47–50) and several studies have also shown signs of efficacy in the treatment of T1D and GvHD (51–56).

**Figure 2:**
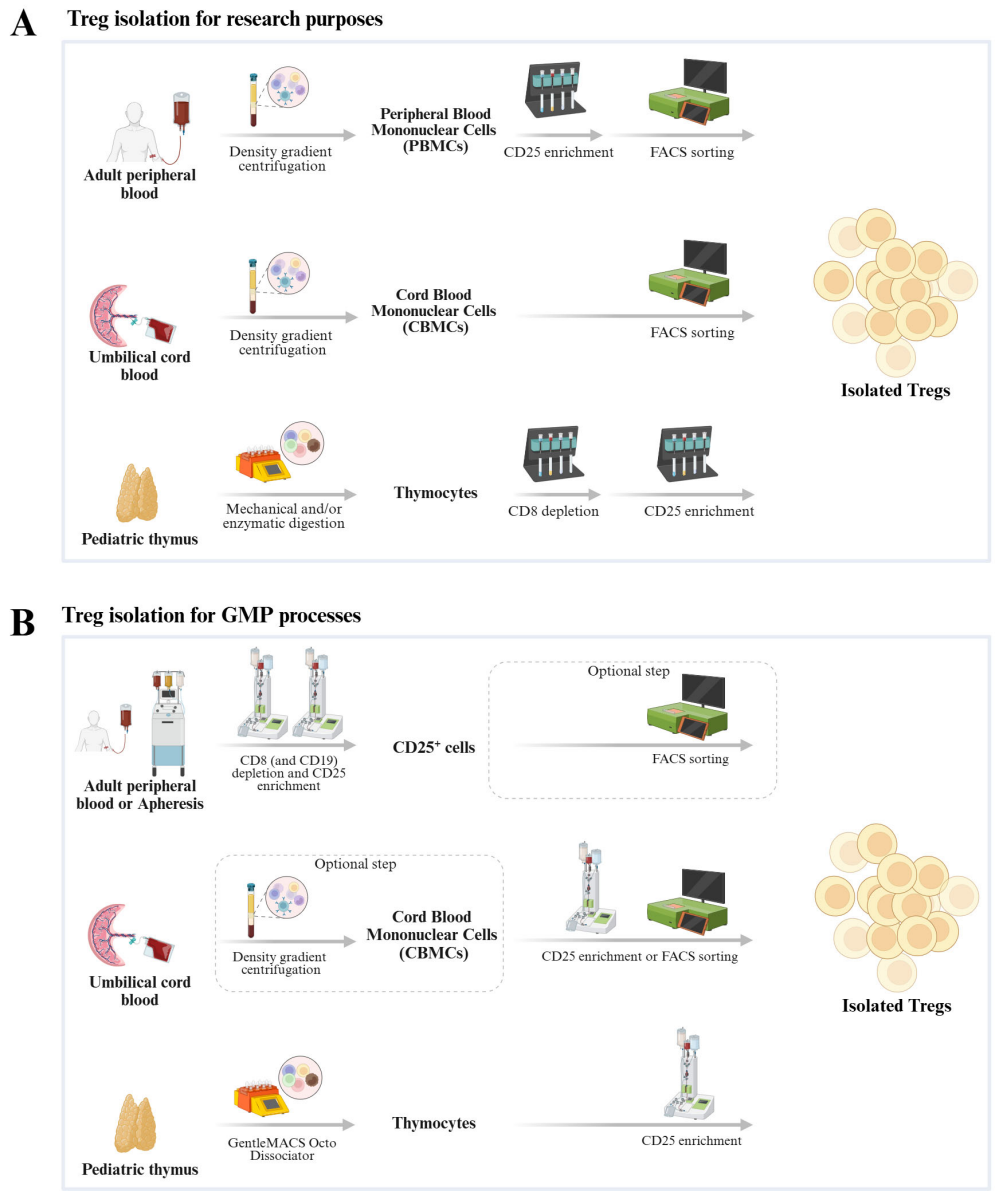
Schematic representation of methods used to isolate Tregs from three different sources for research purposes **(A)** and GMP processes **(B)**.

Treg cells for adoptive transfer are typically obtained from peripheral blood or umbilical cord blood, but some limitations have been hindering their large-scale usage in clinical applications. Adult peripheral blood is one of the most common sources since it can be easily obtained. However, the majority of adult Tregs exhibit an antigen-experienced memory phenotype, which is associated with a risk of phenotypic instability during prolonged *ex vivo* expansion or repeated stimulation, along with an overall low expansion potential (57–59). This also means that their lifespan and consequently their clinical potential are reduced (60, 61). The second prominent Treg source is umbilical cord blood (54, 62–64). Unlike adult peripheral blood, the vast majority of Tregs isolated from cord blood are naïve. However, the number of Tregs that can be obtained from one donation is very low. Thus, a massive *ex vivo* expansion is required to obtain relevant cell numbers for therapy (61, 65–67). Lately, Tregs isolated from pediatric thymi, which are routinely removed during pediatric heart surgeries to allow surgeons adequate exposure to the retrosternal operative field, have shown promising features that overcome the abovementioned limitations of Tregs derived from blood sources and enable the isolation of high numbers of naïve Tregs (60, 61, 68) (**Table 1**; **Figure 2**).

**Table 1:** A brief summary of the advantages and disadvantages of Tregs isolated from three different sources.

	Adult peripheral blood	Umbilical cord blood	Pediatric thymus	References
Maximal Treg isolation yield	10–60 million (whole blood, buffy coat or apheresis)	5–7 million (fresh or frozen cord blood)	200–300 million (average thymus weight of 20 g)	(60, 61)
Abundance of peripherally induced Tregs	High	Low	Low	(61, 69)
Treg antigen experience	Age dependent, memory > naïve phenotype (CD45RO^+^)	Mostly naïve phenotype (CD45RA^+^)	Mostly naïve phenotype	(57, 58, 62)
*Ex vivo* expansion capacity	Moderate	High	Moderate	(54, 60, 64, 70)
Lineage stability (FOXP3^+^)	Unstable	Stable	Stable	(60, 69)
*In vitro* potency	Limited suppressive capacity	High suppressive capacity	High suppressive capacity	(60, 62, 69)
Longevity	More senescent phenotype due to shortened telomeres	Longer telomere length	Longer telomere length	(60, 71, 72)
Immunogenicity	High	Low	Low	(61, 73, 74)

Recently, the first thyTreg studies have moved from the preclinical stage into phase 1/2 clinical trials, including the transfusion of autologous thyTregs to prevent rejection in heart transplantation in children (NCT04924491) and transfusion of allogeneic thyTregs to control the immune dysregulation associated with SARS-CoV-2 infection and/or acute respiratory distress syndrome (NCT06052436). Available early clinical trial data show safety, tolerability and capacity to restore the Treg pool (74).

Similar to the early days of cell therapy with peripheral blood Tregs, reported thyTreg production protocols differ substantially between investigators. There is a need to better understand thyTreg products and to relate them to isolation and expansion strategies which will eventually allow to maximize clinical efficacy. This review is intended to be a first step in this process and summarizes what has been established by different groups regarding the manufacturing procedures of thyTregs, the rationale for their clinical application and the current ongoing clinical trials.

## 2 Isolation of Tregs from thymic tissue

Thymic tissue is collected during pediatric corrective cardiac surgery after parental/caregiver written informed consent and stored in sterile containers with medium or sodium chloride solution supplemented with antibiotics and antimycotics. The tissue is then kept at 2-8°C and transported to the processing site. ThyTreg isolation typically starts with single thymocyte isolation followed by Treg enrichment.

### 2.1 Thymocyte isolation by mechanical and/or enzymatic digestion

Reported digestion procedures for thymic tissue fragments to obtain single thymocytes vary between published protocols based on different digestion media, dissociation machines, and whether mechanical and/or enzymatic cell digestion is used (**Supplementary Table 1**).

The Powrie and Suri-Payer groups described cutting thymic tissue into small pieces and gently pressing them through a metal sieve to obtain a single cell suspension (75, 76). The cells were then washed twice in PBS containing 0.2% bovine serum albumin (BSA) or RPMI medium containing 2% heat-inactivated fetal calf serum (FCS) and HEPES (10 mM). The yield and viability of thymocytes obtained by this method were not reported. Later, Suri-Payer and Rudin, and Teague groups (77, 78) reported obtaining a single cell suspension by gently passing the thymus tissue through a 70 µm nylon sieve.

Other publications reported tissue dispersion by syringe plungers and subsequent separation by Ficoll Paque PLUS density gradient to obtain single thymocytes (36, 37, 79–81). Additional purification procedures were included in one of those studies such as the use of MACS MicroBeads (Miltenyi Biotec) to remove non-thymocytes such as CD56^+^, CD19^+^, CD14^+^ and CD11c^+^ cells resulting in a purity over 95% CD2^+^ cells (79). Others employed negative depletion with a mixture of mouse monoclonal antibodies against the markers CD11c, CD14, CD15, CD20, CD56, and CD235a together with CD8 followed by incubation with goat anti-mouse IgG coated magnetic beads (M-450, Dynal) to obtain CD4^+^ T cell thymocytes (80) or the use of FACSAria (BD) cell sorting using the lineage markers CD14, CD16, CD20, CD56 and HLA-DR combined with CD3, CD4 and CD8 staining to sort human triple negative and CD4 immature single positive thymocytes (81).

In 2016, the group of Levings and West (60) reported the use of RPMI medium (Wisent, Inc.) supplemented with L-glutamine, 1% Penicilin/Streptomycin (Pen/Strep) and 10% heat inactivated fetal bovine serum (FBS) for mechanical digestion of thymus tissue using the gentleMACS Dissociator (Miltenyi Biotec). Large numbers of thymocytes were isolated using this method (925×10^6^ ± 279×10^6^ cells per gram of tissue).

Subsequently, Levings’ group documented a Good Manufacturing Practice (GMP) compliant protocol for thyTreg isolation that aimed to minimize manual steps towards a closed system approach (70). Two different media for tissue degradation were compared: RPMI medium (Thermo Fisher Scientific) with 10% heat-inactivated FBS, 1% GlutaMAX and 1% Pen/Strep or ImmunoCult-XF T cell Expansion Medium (ImmunoCult-XF) (STEMCELL Technologies) with 1% Pen/Strep. They also compared two dissociation methods. One was using manual dissociation employing scissors, razor blades or a McIlwain tissue chopper (Campden Instruments Ltd.), and the other was an automated procedure using the gentleMACS Dissociator. Both methods resulted in a high cell yield, with manual dissociation showing superior thymocyte yield and viability.

In 2022, the Martínez-Bonet and Correa-Rocha group reported that collected thymic tissue was placed in TexMACS GMP medium (Miltenyi Biotec) supplemented with 1% antifungal antibiotic (Pen/Strep/Amphotericin B) and then processed with the gentleMACS Dissociator for mechanical degradation. Afterwards, thymocytes were suspended in PBS/EDTA with 0.5% human serum albumin and transferred to a cell bag until further processing. High yields (540-3,370 ×10^6^ per gram) and viability (96.33% ± 0.99%) of thymocytes were obtained (61).

Different from the above-mentioned method of sole mechanical dissociation, the Lombardi laboratory described the use of additional enzymatic digestion. During their mechanical digestion of thymic fragments in the GentleMACS system, Collagenase (0.2 mg/mL) and deoxyribonuclease I (5 mg/mL) were added to the medium consisting of X-VIVO 15 (Lonza) with 5% HS AB male and amphotericin B (0.5 μg/mL) (68). It was reported that 650 (350-1,080) × 10^6^ thymocytes were obtained per gram of thymus tissue, which is fewer than those obtained by mechanical/manual dissociation. In addition, the Treg yield after isolation was significantly less than with sole mechanical dissociation. (**Supplementary Table 1**). These findings indicate that enzymatic tissue digestion does not enhance thymocyte or Treg recovery. Potential contributing factors include collagenase-mediated cytotoxicity—a parameter not quantified here—and cleavage of surface markers such as CD25, which is critical for Treg identification and isolation.

In general, mechanical dissociation using the GentleMACS Dissociator (Miltenyi Biotec) produces a high yield of viable thymocytes and has been shown to be applicable to GMP-compliant production (**Supplementary Table 1**). However, the dissociation medium varies between different groups. A systematic comparison of media formulations would help to elucidate their influence on thymocyte viability, recovery efficiency, and phenotypic integrity—particularly for downstream applications such as Treg isolation and functional analyses.

### 2.2 Treg isolation from single thymocytes

The most commonly employed surface marker to identify and isolate Tregs from single thymocytes is CD25. Some research groups also included a CD8^+^ cell depletion step in addition to CD25^+^ cell enrichment. In addition to CD25 and CD8, the surface markers CD3, CD4 and CD45RA have been reported for sorting of pure populations and Treg subsets.

#### 2.2.1 Single CD25^+^ magnetic enrichment

The group of Martínez-Bonet and Correa-Rocha reported direct CD25^+^ enrichment of thymocytes to enrich Tregs (61). In their procedure, thymocytes were filtered through a 40 μm pore filter prior to CD25^+^ immunomagnetic isolation using human CD25 MicroBeads II with LS columns on a QuadroMACS Separator (all from Miltenyi Biotec). The number of obtained CD25^+^ cells per gram of thymus was reported to be around 9.96 (1.32-21.59) million, summing up to around 200 million CD25^+^ cells from a single average weighted infant thymus of 20 g. The average frequency of FOXP3^+^ cells within isolated CD25^+^ thymocytes was 67%. Importantly, the authors translated this process to a GMP compliant protocol using CliniMACS CD25 GMP MicroBeads on a CliniMACS Plus instrument (Miltenyi Biotec). Recovery, viability, purity and phenotype of these Tregs were similar to those cells obtained at research scale.

#### 2.2.2 CD8^+^ depletion in addition to CD25^+^ enrichment

Since thymocytes contain a large population of CD4^+^CD8^+^ DP cells, some groups included a CD8^+^ depletion step prior to CD25^+^ cell enrichment to remove CD4^+^CD8^+^ DP and CD8^+^ SP cells to isolate CD4^+^ SP thyTregs.

In 2001, Stephens et al. reported a CD8^+^ depletion step before CD25^+^ enrichment using the “rosette” technique (75, 82) based on sheep red blood cells coated with αCD8 antibody (OKT8), triggering CD8^+^ T cell capture and rosette formation, allowing their removal by density centrifugation (75, 82). Using this protocol, CD4^+^ single positive cells represented 70-80% of the resulting intermediate product. Cells were further enriched for CD25^+^ by incubation with an anti-CD25-FITC antibody and separated by using anti-FITC MACS beads (Miltenyi Biotec). The result shows that the isolated CD4^+^CD25^+^ thymocytes represented 9% ± 1% of total CD4^+^CD8^–^ thymocytes.

In 2016, the group of Levings and West described CD8^+^ depletion by complement mediated lysis, achieved by adding 1 mg/mL mouse α-human CD8a monoclonal antibody and HLA-ABC Rabbit Complement (dilution: 1:30) to the isolated single thymocytes, at a concentration of 20 million cells/mL medium at 37°C for 1h (60) (adapted from (83)). The depleted cell suspension was filtered, washed and resuspended in MACS buffer (PBS supplemented with 0.5% BSA and 2mM EDTA). It was reported that the percentage of CD3^+^CD4^+^CD8^-^ cells increased from around 10% to 46%, and these cells were then further enriched for CD25^+^ using CD25 MicroBeads-II on an autoMACS Pro-Separator (Miltenyi Biotec). After the CD25^+^ selection, 14.1 ± 4.2 x10^6^ CD3^+^CD4^+^CD25^+^ cells per gram of tissue were obtained. The percentage of CD25-expressing cells in the final product was 88.7% ± 6.6% and the frequency of FOXP3^+^ cells within the CD25^+^ cells was 78.9% ± 5.4%.

Later, the Levings group reported the inclusion of CD8^+^ depletion after CD25^+^ enrichment (70, 84). They obtained CD25^+^CD8^−^ cells by CD25 positive selection using Releasable RapidSpheres according to the manufacturer’s instructions (STEMCELL Technologies), followed by negative selection of CD8^-^ cells. They also compared the influence of the two used isolation methods – manual dissociation and the GentleMACS dissociator - on thyTreg frequency, yield, viability and FOXP3 expression levels. The result shows that there are no significant differences in Treg (CD4^+^CD8^-^CD25^+^ cells) frequency within the total live thymocytes. The median Treg recovery from thymocytes was 17.0% (1.4–83.9%) using manual dissociation and 14.1% (5.0–45.3%) using GentleMACS. Treg purity (defined as CD25^+^CD4^+^CD8^−^ cells) was significantly higher after manual dissociation (94.4%, range 80.8–99.1%) than after GentleMACS (87.4%, range 61.7–95.3%). However, the latter method was associated with increased FOXP3 expression.

Similarly, the Lombardi group reported the isolation of the thyTregs by CD8^+^ depletion followed by CD25 enrichment using immunomagnetic beads (Miltenyi Biotec) with a yield of 2.02 × 10^6^ Tregs/g (range 0.59–6.65 × 10^6^), with on average 80% of the cells showing the CD3^+^CD4^+^CD8^–^CD25^+^CD127^–^ Treg phenotype (range 72–90.8%) (68).

#### 2.2.3 Isolation using additional markers

In addition to a single step of CD25^+^ cell enrichment for thyTregs, Martínez-Bonet and Correa-Rocha group reported the usage of the MACSQuant Tyto cell sorter (Miltenyi Biotec) to separate their 7-days post expansion thyTreg product into CD4^+^ SP thyTregs and CD4^+^CD8^+^ DP thyTregs (61). 50 million thyTregs were labeled with CD4 and CD8 antibodies, washed and resuspended at 5 million cells/mL in MACSQuant Tyto Running Buffer. The labeled cells were first sorted for CD4SP cells and the uncollected cells underwent a second round of sorting for CD4CD8 DP cells. By analyzing the demethylation status of the Treg-specific demethylation region (TSDR) of the Foxp3 gene, which indicates the stability of the Treg-specific phenotype, they showed that 94.1% of CD4CD8 DP population exhibited a demethylated TSDR similar to that of CD4SP cells (92.8% demethylation), whereas the total thyTreg population showed 91.6% TSDR demethylation. In addition, they observed that the CD4CD8 DP population could contribute to the high purity and suppressive capacity of their final product. Thus, they came to the conclusion that CD25^+^ enrichment was sufficient for Treg purification from thymocytes.

The Liu’s group documented FACS sorting (FACSAria, BD) of the ICOS^+^ and ICOS^-^ subpopulations of CD4^+^CD8^−^CD25^+^ cells (80). Their results showed that the sorted CD25^+^ICOS^+^ and CD25^+^ICOS^−^ subsets both expressed FOXP3.

The Lombardi group reported FACS sorting (FACSAria, BD) of the CD45RA^+^ subpopulation from their thyTregs, to compare the phenotype, stability, and methylation level of total thyTregs and CD45RA^+^ thyTregs (68). Their results show there is no significant difference between these two populations and suggest that the additional step of isolating the CD45RA^+^ subpopulation is dispensable. It is worth noting that the majority of thyTregs (CD3^+^CD4^+^CD8^-^CD25^+^) are CD45RA^-^ (68). CD45RA expression is reportedly acquired later than CD25 and FOXP3, which are only expressed in mature, naïve Tregs ready to leave the thymus (85).

In summary, CD25^+^ enrichment was consistently applied between investigators, either using MACS beads or by including CD25 in the FACS sorting panel. Additional CD8^+^ depletion steps and the inclusion of further markers result in a more specific and defined cell population.

It is important to note that the identification and isolation of a pure Treg population is difficult due to the lack of a cell surface marker specific for Tregs (20, 60, 86, 87). The most widely used phenotype panel CD4^+^/CD25^hi^/CD127^lo^/FOXP3^+^ may be the most specific way to identify mature thyTregs. Since the important Treg marker FOXP3 is localized intranuclearly (88, 89), flow sorting with CD4^+^CD8^-^CD25^hi^CD127^lo^ gating can be applied to obtain pure mature CD4^+^ thyTregs. Further research to identify novel specific surface marker(s) to detect and isolate Tregs from thymocytes with high purity, and to understand the subsets that are in different developmental states and their specific function are of high importance to the field.

## 3 *Ex vivo* culture and expansion of thymus-isolated Tregs

### 3.1 Early reports on thyTreg culture

Two initial studies have used divergent methods to culture thyTregs (**Supplementary Table 2**). In 2001, Stephens et al. reported research grade culture of CD4^+^CD25^+^ thyTreg cells in RPMI with 10% heat-inactivated FCS, supplemented with Pen/Strep, glutamine, sodium pyruvate and 2-mercaptoethanol in U-bottom 96-well plates (75, 82). In most experiments within this study, gamma irradiated CD8-depleted thymocytes were added as a source of antigen-presenting cells. Cells were stimulated with phytohemagglutinin-L (Sigma), concanavalin A or with phorbol-12-myristate-13-acetate and ionomycin. In some experiments, recombinant human IL-2 (Boehringer Mannheim) was added to the cultures at a final concentration of 20–100 IU/mL. After culturing for 72 h, cells were pulsed with 0.5 µCi [^3^H] thymidine and cultured a further 18 h before harvest. These early experiments indicated that CD4^+^CD25^+^ thyTreg proliferate rather poorly but are able to suppress proliferation of other CD4^+^ cells *in vitro*.

In 2008, Ito et al. reported a ten-day culture protocol of sorted CD4^+^CD8^−^CD25^+^ICOS^+^ (ICOS^+^ Tregs) and CD4^+^CD8^−^CD25^+^ICOS^−^ (ICOS^-^ Tregs) thymocytes (80). They applied two rounds of 5-day stimulation using L cells (a mouse fibroblast cell line) or ICOSL expressing L cells pre-coated with anti-CD3 antibody (0.2 μg/mL) and cultured the cells in RPMI 1640 supplemented with 10% FCS, 2 mM L-glutamine, 1 mM sodium pyruvate, penicillin G, streptomycin, 50 IU/mL of IL-2 and 20 ng/mL of IL-7 (R&D Systems). The results suggest that the sorted ICOS^+^FOXP3^+^ Treg cells showed a higher percentage of IL-10^+^ cells than the ICOS^-^FOXP3^+^ Treg cells after 10 days of culture; while the ICOS^-^FOXP3^+^ Tregs expressed higher levels of membrane-bound TGF-β1 than the ICOS^+^FOXP3^+^ Tregs on day 5 of culture. Both cultured subsets had the ability to suppress the proliferation of autologous CD4^+^CD25^-^ T cells stimulated by allogeneic monocyte-derived DCs. Survival and proliferation of these two thyTreg subtypes was achieved by co-stimulation with ICOSL or ICOSL and anti-CD28 antibody respectively, while total fold expansion was not reported.

### 3.2 Treg dosage for adoptive therapy

Even though the minimum number of Tregs needed to show efficacy in cell therapy is not yet known, initial case studies using polyclonal expanded Tregs derived from stem cell donor peripheral blood and apheresis infused 0.10-4.45 x10^6^ cells/kg body weight to GvHD patients (51, 53).

Later, 10–30 million autologous Tregs/kg infused to T1D patients were reported (52, 90) and another publication reported that doses of 5-2,665 million blood-derived polyclonal Tregs in total were infused to patients with T1D (47). In 2024, it was reported that the T1D patients in a phase 2 clinical trial received either a high dose of 20 million cells/kg or a low dose of 1 million cells/kg (50). Other reports show that up to 10 million/kg autologous peripheral blood Tregs were infused into patients before or after receiving a liver transplant in two recent studies (55, 91). The ONE study addressed 0.5–10 million autologous Tregs/kg given after kidney transplant and showed no safety concerns (48).

In addition, two independent studies using third party umbilical cord blood Tregs reported a single dose of 1 million cells/kg (92) or 3 million cells/kg (93), respectively, to prevent GvHD in hematopoietic stem cell transplant patients. Another clinical trial investigating infusion of umbilical cord blood Tregs showed that treatment with a single dose of 3–30 million cells/kg or two doses with 100 million cells/kg in total after double umbilical cord blood transplantation was safe and resulted in a low risk of acute GvHD in patients with advanced hematological cancer or other disorders (54).

Overall, among the above-mentioned therapies, even 100 million/kg of third party cord blood-derived Treg have been shown to be safe, and considering an average-weighted adult of 70kg, the target dose of Tregs would be 7,000 million cells. According to the previously mentioned thyTregs isolation, around 200–300 million cells can be obtained from a single average weighted infant thymus (60, 61). This means that a 30-fold expansion of thyTreg could be aimed at for clinical translation.

### 3.3 ThyTreg *ex vivo* expansion techniques

To optimize thyTreg numbers towards therapeutic applications, several groups aimed at expanding isolated Tregs and different expansion protocols have been reported (**Supplementary Table 2**).

In 2016, the groups of Levings and West published a method to expand thyTregs (60). Tregs were stimulated with irradiated (40 Gy) L cells (expressing human CD80, CD32 and CD58) loaded with mouse α-human CD3 monoclonal antibody (60, 84, 94) at a 1:1 ratio in culture medium (OpTmizer T-Cell Expansion Serum-free Medium (Life Technologies) supplemented with l-Glutamax, 1% Pen/Strep, IL-2 (1000 IU/mL) and 100 ng/mL rapamycin). On day 7, thyTregs were re-stimulated with α-CD3-loaded L cells and the culture medium was changed to the medium without rapamycin. On day 11, the expanded cells were washed and rested overnight in expansion medium with 1000 IU/mL IL-2. The results show that the expanded cells had a typical Treg phenotype, stable FOXP3 expression, unchanged telomere length, high suppressive capacity *in vitro* and *in vivo* and remained stable and suppressive under polarizing conditions. Their proliferation rate was considered slow by the authors, with an expansion ranging from 2–9 fold after 7 days of culture. However, cell restimulation and refreshment of the culture medium without rapamycin increased cell expansion to 6–33 fold by day 11. The slow expansion rate might be explained by the used OpTmizer medium. The same investigators later reported a 300-fold expansion by day 12 using the same stimulation method but ImmunoCult-XF medium. (70). Due to the increased process and regulatory complexity for a manufacturing process using artificial antigen-presenting cells (aAPC), some groups seek cell-free alternatives. In 2019, a study from the Levings’ group reported the comparison of cell-free alternatives using aAPCs for thyTreg expansion (70). In this report, four different cell-free activation reagents were compared, with respective media: ImmunoCult CD3/CD28 T Cell Activator with ImmunoCult-XF medium (StemCell Technlologies), ImmunoCult CD3/CD28/CD2 T Cell Activator with ImmunoCult-XF medium (StemCell Technlologies), Dynabeads Treg Xpander (Thermo Fisher Scientific) with X-Vivo 15 (Lonza) with 5% CTS Immune Cell Serum Replacement (Thermo Fisher Scientific), and T Cell TransAct with TexMACS (Miltenyi Biotec) with 5% human serum. The aAPC condition using ImmunoCult-XF medium was included for comparison. In all conditions, thyTregs were seeded at 0.5 ×10^6^/mL in 96-well or 24-well plates, media/additives were refreshed every 2–3 days and cells were passaged keeping the cell concentration constant. ThyTregs were activated on day 0, restimulated on day 7, and harvested/analyzed on days 12 and 15. Rapamycin was included in the culture medium from day 0–7 during thyTreg expansion. Recombinant human IL-2 (Proleukin) (1000 IU/mL) was added from day 0. Their results showed that the aAPC based approach led to the highest fold expansion on both days 12 and 15, while T cell TransAct exhibited the lowest expansion. However, cells activated with aAPC showed lower viability on day 15. Their phenotype analysis of the cells from day 12 show that naïve markers remained high for all the conditions, while some other activation markers were up or down-regulated. FOXP3 expression was similar for every condition. From this test, two cell-free activation agents, Treg Xpander and CD3/CD28/CD2 T Cell Activator, gave the highest cell fold expansion and FOXP3 expression and were therefore selected for further medium comparison. Overall, thyTreg expansion in ImmunoCult-XF with Treg Xpander stimulation with or without serum replacement showed the highest fold expansion (317 and 295-fold, respectively), viability and FOXP3 expression levels after 15 days culture. As cell growth, viability and FOXP3 expression were not significantly affected by serum replacement, the ImmunoCult-XF without serum replacement and Treg Xpander stimulation was thus used for their following experiments.

Another Treg expansion approach was published by the group of Lombardi (68). After CD25^+^ magnetic enrichment and cell sorting for the CD45A^+^ Treg subpopulation, the cells were cultured for 36 days in X-Vivo 15 medium, supplemented with 5% HS AB, IL-2 (1000 IU/mL), with or without rapamycin (100 nM). The MACS GMP ExpAct Treg Kit (Miltenyi Biotec) (1:1 bead to cell ratio) was used for stimulation. Every 2 days after initial stimulation, rapamycin and IL-2 were refreshed. Cells were restimulated every 10–12 days and harvested on day 36. The results showed the 36 days of expansion resulted in high expansion (>900 fold) and high viability (>90%), and no significant differences in phenotype, stability and TSDR methylation status between total bulk Tregs and the CD45A^+^ subpopulation. It is also reported that IFN-γ-producing cells were only detected in Tregs expanded in the absence of rapamycin, suggesting the importance of rapamycin in culture medium to obtain a stable Treg product suitable for cell therapy.

In 2022, the group of Correa-Rocha (61) reported that the isolated CD25^+^ cells were cultured in TexMACS GMP medium (Miltenyi Biotec), with 600 IU/mL IL-2 at 1 million cells/mL in a 24-well plate. T cell TransAct (Miltenyi Biotec) was added according to manufacturer’s instruction at the starting of culture to stimulate thyTregs and promote their activation and expansion. After 3 days of culture, half of the medium was replaced with fresh medium. Cells were monitored on the following three days, and passaged to keep the cell concentration around 1 million cells/mL. Cells were harvested at day 7. After the 7-days culture, cells had expanded 6.9-fold, and the harvested cells presented very high viability (92.41%) and purity (95.2% of CD25^+^FOXP3^+^ cells).

In the same report, the authors also described a GMP compliant protocol. ThyTreg cells were cultured in TexMACS GMP medium supplemented with MACS GMP IL-2 (600 IU/mL, Miltenyi Biotec) and MACS GMP T cell TransAct (according to manufactuer’s instruction, Miltenyi Biotec) in 175 cm^2^ flasks. Total thyTregs of 197.8 (61.9-582.9) × 10^6^ cells were seeded and passaged at a concentration of 0.5 million cells/cm^2^. After 7 days of expansion, thyTregs reached an average of 1,230 (532–2,512) × 10^6^ cells with high viability (96.58%) and high purity (83.65% of CD25^+^FOXP3^+^ cells), comparable to thyTregs obtained in the laboratory. In addition, GMP thyTregs showed similar phenotype, cytokine secretion pattern, high suppressive capacity, high levels of TSDR demethylation and stability under proinflammatory conditions when compared to laboratory thyTregs, suggesting successful adaptation and scaling of the protocol to GMP conditions.

Taken together, despite variations in expansion methods between different groups, all studies used a complete culture medium supplemented with IL-2 that promote Treg expansion. A stimulation reagent was used on the initial culture day and some studies also included a re-stimulation(s). All final ThyTreg products showed high viability and purity, and high suppressive capacity demonstrating successful *in vitro* expansion. However, it has been reported that rapamycin supplementation in the culture medium reduces IFN-ɣ producing cells after thyTreg expansion (68), and groups of scientists have reported that a second restimulation is more likely to promote Treg instability and the proliferation of other T cell populations (57, 66). Further optimization of the reported expansion techniques is needed to achieve high expansion while maintaining high purity and potency. It is also relevant to note that cryopreservation of those expanded cells is an attractive option towards an “off-the-shelf” product. It has already been shown that cryopreservation procedures, medium, and time affect thyTreg quality after thawing (70). More studies are required to develop optimal clinical grade cryopreservation methods for thyTregs manufactured with different protocols.

### 3.4 *In vivo* Treg expansion

Building on the crucial role of IL-2 for Treg survival, expansion and function, researchers have sought to identify a therapeutic strategy that leverages IL-2 to enhance endogenous Treg populations, thereby mitigating excessive immune responses. To date, several clinical studies have reported that low-dose IL-2 results in durable clinical improvement in autoimmunity and GvHD and is well tolerated (95–98). Infusions of expanded autologous peripheral Tregs together with IL-2 injections have been reported to be safe and may enhance the suppressive functions of Tregs in individuals with Amyotrophic Lateral Sclerosis, thereby slowing disease progression (99). Other reports on low dose IL-2 treatment following polyclonal Treg therapy in cGvHD did not show clear additional effects (53), whereas a phase I study combining autologous *ex vivo* expanded polyclonal Tregs and low-dose IL-2 in patients with recent-onset T1D has been reported to enhance Treg survival and expansion. However, the study also reported substantial increases in many other immune cell subsets, including NK cells, mucosal-associated invariant T cells, and clonal CD8^+^ T cells (100). Therefore, the off-target effects of low-dose IL-2 must be carefully considered. Building on advances in understanding the molecular interactions between IL-2 and its receptor, researchers have endeavored to design and engineer novel IL-2 molecules that target the Treg population more specifically, such as IL-2 antibody complexes, muteins and fusion proteins (101, 102). Ongoing phase I clinical trials will identify best-in-class molecules and computational artificial intelligence methods are likely to accelerate future developments (102, 103).

## 4 Clinical relevance of thymus-isolated Tregs

Obtaining Tregs from peripheral blood requires the extraction of large volumes of blood to achieve a sufficient number of cells. This is clearly a major limitation for paediatric patients and disease and treatment related immunocompromised subjects. However, the new approach of isolating Tregs from thymus tissue could overcome this challenge.

Large parts of the thymus are routinely removed during pediatric corrective heart surgeries. These removed thymic tissues can be donated for research purposes when the parents or caregivers have signed the written informed consent according to the declaration of Helsinki (36, 37, 60, 61, 68, 70, 85, 104). This means that thymus collection does not involve any additional risks or expenses for patients or physicians, which enhances its potential as a cell source for clinical application.

Some authors claim a number of advantages of thyTregs compared to peripheral blood-obtained Tregs (**Table 1**). First of all, the number of Treg cells that can be obtained from a single infant thymus is higher than the number of Tregs present in the peripheral blood of an average-sized adult (105). This means that although the expansion rate of thyTregs is lower than that observed for peripheral blood Tregs, likely due to their early developmental state, the *ex vivo* expansion protocols required may be shorter to obtain a sufficient number of cells for clinical treatment (60).

Moreover, in comparison to the widely used strategy to obtain pure Treg cells from peripheral blood under GMP conditions using CliniMACS CD8^-^CD25^+^ isolation and sorting using the markers CD3^+^CD4^+^CD25^hi^CD127^-^, it is reported that a single step of magnetic enrichment of CD25^+^ cells can isolate relatively pure thyTreg cells from the thymocytes (61), which reduces the manufacturing complexity and increases cell yield, although further studies are needed to address the function of the sub-populations such as CD4^+^CD8^+^ DP thyTregs.

In addition, thyTregs have longer telomeres than adult blood Tregs, and the telomere length does not seem to alter after *ex vivo* expansion. This suggests that thymus Tregs can undergo more cell division cycles after isolation and have better *in vivo* survival and functional capability (60, 71, 72).

Several studies have shown that not all CD25^+^ cells express FOXP3 right after isolation of Tregs from the thymus, since its expression usually starts at a later phase during Treg development from thymocytes (60, 85, 106). However, after expansion, these cells present stable FOXP3 expression levels as well as other typical Treg features such as demethylated TSDR and high expression levels of CD25, Helios and CTLA-4. They also maintain their stability and suppressive features under pro-inflammatory conditions, in contrast to adult blood Tregs, which typically present a more variable response, indicating phenotypic instability (60). Although those features are similar to cord blood derived Tregs, the quantity of Tregs that can be isolated from a single infant thymus is 50–100 times higher than the number of Tregs that can be obtained from a cord blood donation.

Summing up, thyTreg cells are abundant, have an increased lifespan, great suppressive capacity, stability and potency (**Table 1**). The higher quantity and potency allow for several therapeutic doses from a single thymus, with the possibility of cryopreservation. This enables thymus tissue to be used as an alternative source of Tregs for therapeutic applications with superior potential for allogeneic applications.

Allogeneic cell therapy offers several advantages compared to autologous treatment. Successes have been reported for allogeneic peripheral blood Tregs transplantation in HLA-haploidentical setting to counteract the acute and chronic GvHD potential of a high number of donor conventional T cells in adults (107), and for allogeneic cord blood Tregs transplantation in HLA-partial match to prevent acute GvHD in adults (54, 93). Allogeneic adoptive transfer of Tregs is based on the knowledge that Tregs are potent suppressors of allogeneic T effector cell proliferation *in vitro*. Furthermore, *ex vivo* expansion of Tregs uses polyclonal stimulation with antibodies directed against CD3 and CD28 mimicking antigen-presenting cells. Once activated, Tregs suppress in a bystander manner which renders them functional after infusion. It was further shown that HLA disparities do not affect Treg suppressive function in early stages after Treg infusion which demonstrated that the use of third-party Tregs is a valuable alternative to stem cell donor-derived Treg immunotherapy in suppressing acute GvHD (108). However, it was shown that third-party Tregs survive for a shorter period of time *in vivo* than donor-type Tregs (109).

Although the generation of a thymocyte bank which would allow HLA matching similar to cord blood banks is theoretically feasible given the abundance of discarded thymi, it has to our knowledge not yet been envisioned. One of the reasons may be the postulated low immunogenicity compared to expanded blood derived Tregs (73). ThyTreg were reported to present lower levels of HLA-ABC and HLA-DR, even though HLA-ABC expression was reported to be similar between thymic, infant blood and adult blood Tregs directly after isolation (73). The Correa-Rocha group also addressed the undifferentiated character of thyTregs which could confer hypoimmunogenic properties (61, 74), suggesting that thyTregs would be less likely to be rejected in an allogeneic setting and may carry a low risk of adverse effects. Therefore, thymus tissue may very well represent a superior source towards the development of “off the shelf” allogeneic Treg cell therapy approaches.

## 5 Clinical applications using thymus-isolated Tregs

In 2022, Bernaldo-de-Quirόs et al. reported a GMP protocol to produce thyTreg cells from pediatric thymic tissue (61). Their developed thyTreg product has been approved by the Spanish Drug Agency (AEMPS) to be administered as cell therapy. In 2020, a phase 1/2 clinical trial (NCT04924491) was initiated to evaluate safety and efficacy of their autologous thyTreg product (THYTECH1) to prevent rejection in heart transplant children. In 2023, a 2-year follow-up of the first patient treated with the autologous thyTregs undergoing heart transplantation within the trial was published (74). The case report compared the patient (aged 7 months) who received autologous thyTregs that had been *ex vivo* expanded for 7 days post-operatively, with four other control patients who did not receive thyTregs (age <2 years, mean age = 6.25 months at transplantation). All patients underwent thymectomy (estimated >90% thymic tissue resection) before heart transplantation. The patient treated with thyTregs was infused 9 days after thymectomy and heart transplantation, upon stabilization from surgery. It is reported that there were no adverse effects that could be attributed to thyTreg administration. Additionally, the Treg frequency within CD4^+^ T cells in the periphery of the patient treated with thyTregs remained higher than pre-transplant levels throughout the 2-year follow-up period, while the Treg frequency in the control children was lower than the pre-treatment levels from 9 months post-transplant and reached levels 40% lower than pre-transplant levels by the end of the follow-up period. These initial results suggest that autologous thyTreg therapy is safe and has the capacity to restore the Treg pool. However, autologous Treg therapy still has the drawbacks of high cost, long vein-to-vein procedures, treatment time inflexibility and reduced Treg efficacy due to immunosuppressive regimens. In contrast to peripheral blood or cord blood as a source, autologous therapy with ThyTreg is limited to indications where heart surgery is warranted in the pediatric patients.

As allogeneic Treg cells can be generated from healthy donors with the possibility of cryopreservation of large batches for *ad hoc* treatment, allogeneic approaches would have many benefits for patients. 14 clinical trials using allogeneic Tregs derived from different sources for the treatment of various diseases including GvHD, hematologic malignancies, COVID-19-related ARDS, myelofibrosis and amyotrophic lateral sclerosis (ALS) were recently summarized by Chmiel et al. (110), including some third party approaches. Other trials include mainly stem cell donor derived personalised therapies (NCT06551584, NCT06845592, NCT06864598). However, there is also a third-party allogeneic Treg trial for the prevention of GvHD currently underway (EudraCT 2021-006490-32). In 2022, THYTECH was founded as a spin-off from the University General Hospital Gregorio Marañón in Madrid. They are committed to develop “off-the-shelf” regulatory T-cell therapies for patients with severe immunological and inflammatory disorders using their thyTreg products. In 2023, THYTECH initiated a phase 1/2 clinical trial to evaluate the safety and efficacy of the allogeneic thyTregs (THYTECH2) in controlling the immune dysregulation associated with SARS-CoV-2 infection and/or acute respiratory distress syndrome (NCT06052436).

The two mentioned studies with thyTregs provide the first evidence for the feasibility of using thyTregs in both autologous and allogeneic settings to prevent transplant rejection or treat immune dysregulation in both children and adults. These seminal studies together with planned clinical trials in further allogeneic settings will shed additional light into the *in vivo* immunogenicity through monitoring of Treg persistence, adverse events and efficacy.

## 6 Conclusion and outlook

Tregs are very important to regulate exacerbated immune responses, maintain immunological tolerance, and prevent from immune-related complications, such as graft-versus-host disease, transplant rejection, and autoimmune disorders. Due to their suppressive ability and their potential for promoting tissue homeostasis and repair, clinical trials using autologous or allogeneic Tregs as adoptive cellular therapy have demonstrated safety and suggested efficacy in preventing and ameliorating GvHD after hematopoietic stem cell transplant, and delaying progression of autoimmunity.

Compared to the Tregs obtained from the most common sources peripheral blood and umbilical cord blood, thymus-derived Tregs have certain advantages, mainly due to their antigen unexperienced state and low immunogenicity, the higher cell number that can be obtained and simple isolation procedures. Different publications have described a number of Treg isolation and expansion protocols over recent years, and several approaches have revealed promising results for clinical translation. However, cells isolated using the abovementioned methods contain thyTregs at different developmental stages. Their differentiation and phenotype after *in vitro* expansion are not yet completely understood and further studies are critical. Additionally, specific markers to isolate more homogenous mature thyTregs are needed considering the advancement of Treg therapy towards targeted approaches such as chimeric antigen receptor (CAR)-Treg requiring pure well defined populations for genetic engineering.

Moreover, it is necessary to develop optimized expansion conditions to obtain thyTreg products with high purity, viability and functionality for the clinical applications of the future.

To date, no study has been conducted on *in vivo* stimulation strategies such as low dose IL-2 or IL-2 designer molecules in conjunction with adoptive transfer of thyTregs. The potential for *in vivo* expansion may ultimately eliminate the need for *ex vivo* culture. Given the high Treg isolation yield and reduced immunogenicity, pediatric thymus –a discarded byproduct of surgical procedures- presents an attractive candidate for isolating defined Treg subsets, which could be leveraged in combination therapies and targeted immunotherapeutic approaches.
